# Clinical characteristics of *Haemophilus ducreyi* genital ulcer disease in Malawi

**DOI:** 10.21203/rs.3.rs-8683388/v1

**Published:** 2026-01-29

**Authors:** Mitch M. Matoga, Jane S. Chen, Sarah E. Rutstein, Gomezgani Lukhanda, Farhang Aghakanian, Jonathan J Juliano, Jonathan B. Parr, Magnus Unemo, Irving F. Hoffman, Arlene C. Sena

**Affiliations:** University of North Carolina Project-Malawi, Lilongwe, Malawi; Institute for Global Health and Infectious Diseases, University of North Carolina, Chapel Hill, USA.; Department of Medicine, University of North Carolina at Chapel Hill, Chapel Hill, NC, USA; Institute for Global Health and Infectious Diseases, University of North Carolina, Chapel Hill, USA.; University of North Carolina Project-Malawi, Lilongwe, Malawi; Institute for Global Health and Infectious Diseases, University of North Carolina, Chapel Hill, USA.; Department of Medicine, University of North Carolina at Chapel Hill, Chapel Hill, NC, USA; Institute for Global Health and Infectious Diseases, University of North Carolina, Chapel Hill, USA.; Department of Medicine, University of North Carolina at Chapel Hill, Chapel Hill, NC, USA; Institute for Global Health and Infectious Diseases, University of North Carolina, Chapel Hill, USA.; WHO Collaborating Centre for Gonorrhoea and Other STIs, Örebro University, Örebro, Sweden; Institute for Global Health, University College London (UCL), London UK.; Department of Medicine, University of North Carolina at Chapel Hill, Chapel Hill, NC, USA; Institute for Global Health and Infectious Diseases, University of North Carolina, Chapel Hill, USA.; Department of Medicine, University of North Carolina at Chapel Hill, Chapel Hill, NC, USA; Institute for Global Health and Infectious Diseases, University of North Carolina, Chapel Hill, USA.

**Keywords:** Haemophilus ducreyi, chancroid, genital ulcer disease (GUD), clinical and sexual characteristics, epidemiology

## Abstract

**Background:**

Chancroid, a sexually transmitted infection (STI) caused by *Haemophilus ducreyi* resulting in genital ulcer disease (GUD), is now considered rare in many parts of the world. However, chancroid has remained highly prevalent in Malawi since the 1990s.

**Methods:**

We combined data from two recent studies conducted in Malawi (2019–2022, 2021) that screened and enrolled patients ≥ 18 years of age presenting to a STI clinic with GUD. Polymerase chain reaction (PCR) was conducted for *H. ducreyi* and other STIs from ulcer swabs. We evaluated demographic, sexual and clinical characteristics of participants with positive *H. ducreyi* PCRs to describe the epidemiology of chancroid using descriptive statistics.

**Results:**

Among 618 participants with GUD, 137 (22%) tested positive for *H. ducreyi* by PCR. Of these, most were male 85 (63%), the median age was 27 years (interquartile range [IQR]: 23, 33) and 19 (17%) had HIV co-infection. About a third (n=42, 31%) were co-infected with other STIs. Among 15 (11%) enrolled participants with additional clinical data, most reported one sexual partner in the past month (median = 1 [IQR: 1, 1). However, a history of prior transactional sex was reported by 5/15 (33%). The clinical presentation of the ulcers varied and over half presented with multiple ulcers (53%). Most ulcers (89%) were associated with pain, but few (20%) had associated inguinal lymphadenopathy.

**Conclusions:**

This review confirmed the high prevalence and persistence of chancroid in Malawi. However, additional investigations are needed to further characterize the epidemiology of chancroid and determine the reasons for its persistence in Malawi.

## SUMMARY

This review confirms the high prevalence and persistence of genital ulcer disease in Malawi and the clinical characteristics of contemporary chancroid infections.

## INTRODUCTION

Chancroid is a sexually transmitted infection (STI) caused by *Haemophilus ducreyi* and typically presents with painful genital ulcers and inguinal lymphadenopathy.^1^ Chancroid is known to facilitate transmission or acquisition of HIV by up to 300-fold per episode of unprotected vaginal intercourse.^2^ Globally, there has been a sustained reduction in the proportion of genital ulcer disease (GUD) caused by *H. ducreyi*,^3^ making it a rare cause such that syndromic management guidelines for GUD in most parts of the world have removed empiric therapy for chancroid.^2^ Since the early 2000s, chancroid has been absent in North America and Europe and very rarely identified in formerly highly prevalent areas such as Africa, Asia, and the Caribbean.^1^

To our knowledge, only two countries in Africa have reported continued high prevalence (> 6%) of chancroid after 2001. In Malawi, *H. ducreyi* has been consistently identified as a frequent cause of GUD among adults presenting to STI clinics; 26% in 1994,^4^ 15% in 2013,^5^ 18% in 2023,^6^ and 23% in 2025.^3^ In South Africa, there are varying rates of chancroid across the country among adults presenting to primary healthcare facilities ranging from 0.5% from 2007–2015 in Johannesburg^7^ to 8.6% from 2018–2019 in the Eastern Cape.^8^ However, neighboring countries like Zambia^9^ and Zimbabwe^10 9^ reported no cases in 2012 and 2018, respectively. The sustained reduction in chancroid cases worldwide has been attributed to mass antimicrobial prophylaxis of high-risk populations and syndromic management in low-to-middle income countries (LMICs).^1,2^

Chancroid can be diagnosed clinically, with classical ulcers (also known as “soft chancres”) typically described as deep, painful ulcers with nonindurated ragged borders; they are also associated with suppurative inguinal lymphadenopathy/buboes in 10–40% of cases.^1^ However, atypical presentations have been noted, and coinfections of chancroid with other etiologies of GUD such as *Treponema pallidum* subspecies *pallidum* and herpes simplex virus (HSV) may affect the clinical presentation.^1^ Based on a systematic review and meta-analyses of the literature, the pooled sensitivity and specificity for chancroid using clinical diagnosis was only found to be 71.9% and 53.1%, respectively.^11^

Due to the limitations in clinical and laboratory diagnoses, the epidemiology of chancroid is poorly defined, highlighting a research gap.^1,2^ Thus, further research is warranted to understand reasons for the high prevalence and persistence of chancroid in Malawi. We therefore conducted a review of two prevalence studies on GUD that identified *H. ducreyi* infections in Malawi, in attempt to describe the demographic, sexual, and clinical characteristics associated with endemic chancroid.

## MATERIAL AND METHODS

### Overview

This review combined data from two recent studies conducted in Malawi that detected *H. ducreyi* as an etiological cause of genital ulcers and provided metadata on demographic, sexual and clinical characteristics.

### Study population

The first study by Chen et al,^6^ recruited men and women ≥18 years of age who presented with GUD to Bwaila District Hospital STI clinic in Lilongwe, Malawi between May and October 2021. Eligible patients had confirmed genital ulcer(s) on examination defined as moist and unhealed ulcer(s). Patients who reported antibiotic use within 30 days of enrollment were excluded. Enrollment was stratified based on HIV status and sex, with an aim to recruit approximately 40% of people living with HIV coinfected with genital ulcers to explore differences in genital ulcer etiology and clinical characteristics by HIV status.^6^

The second study by Matoga et al,^3^ recruited men and women ≥18 years of age presenting with wet/moist anogenital lesions suspected of primary syphilis at Bwaila District Hospital STI clinic in Lilongwe, Malawi between November 2019 and April 2022. Participants were initially screened for syphilis and had ulcer exudate swabs collected for darkfield microscopy and GUD testing. Only participants with confirmed primary or secondary syphilis by darkfield microscopy and rapid point of care (POC) treponemal antibody testing (Alere Determine Syphilis Tp, Abbott) were further enrolled in the study.^3^ Although the recruitment periods between the two studies overlapped, participants could only enroll to one study.

### Study procedures

Both studies collected data on demographics and HIV status on all participants who underwent screening procedures. Participants who were enrolled in the studies had additional collection of medical and sexual histories, STI signs and symptoms and treatment using questionnaires. Enrolled participants received a physical examination and had high-definition photographs of the ulcers taken. Ulcer characteristics (i.e. description of appearance such as borders, surface, size [calculated as a product of bi-dimensional diameters], pain/tenderness and discharge) were documented following a review of notes and photographs by investigators. Lesion/ulcer exudate swabs were collected prior to standard syndromic treatment for GUD, which included benzathine penicillin, acyclovir and ciprofloxacin. Participants with multiple ulcers either had swabs collected from the largest ulcer with each distinct characteristic and/or location^6^ or had swabs collected for up to four distinct ulcers.^3^ Blood samples were collected for *T. pallidum* serologic testing. All participants received counseling and rapid HIV testing according to the Malawi Ministry of Health guidelines.^12^

### Laboratory Testing

DNA was first extracted from all lesion swabs using a Qiagen DNEasy Blood & Tissue kit (Qiagen, Germany) and stored at − 80°C. Molecular testing for STI etiologies including *H. ducreyi* was conducted on stored swabs at the University of North Carolina (UNC) in Chapel Hill, NC, USA.

In the study by Chen, et, al., each sample was tested using real time PCR assays targeting *H. ducreyi, T. pallidum*, HSV-1 and − 2 and *Chlamydia trachomatis* (for *lymphogranuloma venereum* [LGV] detection) on the Atila 9600 PowerGene Plus (Atila Biosystems, USA) machine with positive and negative controls. All samples with amplification above the auto-generated baseline were considered positive. Details of the molecular assays have been previously provided.^6^

In the second study, STI etiology testing also included quantitative *T. pallidum polA* PCR (qPCR) for syphilis^13^ and singleplex PCRs for *H. ducreyi*, HSV-1/2, and *C. trachomatis* using published assays.^3^ Samples with > 0 copy numbers by qPCR were considered positive for T. *pallidum*; samples were considered positive for *H. ducreyi*, HSV, or *C. trachomatis* if the PCR cycle threshold was > 0 and < 40 (supplemental material Tables 2 and 3). Amplicons underwent bidirectional Sanger sequencing at Eton Bioscience (Research Triangle Park, NC, USA) and consensus sequences were deposited in National Center for Biotechnology Information (NCBI) with accession numbers available as supplementary material in this article.^3^ A subset of samples^3^ was sent to the WHO Collaborating Centre for Gonorrhoea and other STIs in Örebro, Sweden, for external validation using the commercially available Allplex Genital Ulcer real-time PCR assay (Seegene Inc, Seoul, South Korea) assay. Samples sent for external validation included (1) all *H. ducreyi* positive samples; (2) all *C. trachomatis* positive samples; and (3) all samples that were negative for pathogens tested.

### Statistical analysis

Statistical analysis was limited to participants with positive chancroid results based on molecular testing (PCR) from the two studies. Descriptive statistics were used to summarize basic demographic characteristics (age, sex, HIV status and co-infection) on screened participants, and additional sexual and clinical characteristics from enrolled participants. Sexual history included number of sexual partners, transactional sex and alcohol or drug use. Clinical characteristics included number of ulcers, ulcer duration, location, association with adenopathy, rash, pain and other STI symptoms. All analyses were performed using SAS v9.4 (Cary, NC, USA) or R v.4.2.2 (Vienna, Austria).

### Ethical considerations

Both studies received ethics approval from the Malawi National Health Sciences Research Ethics Committee and the UNC Institutional Review Board. All participants provided consent before participation.

## RESULTS

From 618 participants with GUD who were screened and enrolled from the two studies, there were 137 (22%) who tested positive for *H. ducreyi* by PCR. Of the 137 chancroid cases, most were male 85 (63%), the median age was 27 years (interquartile range [IQR]: 23, 33) and 19 (17%) had HIV co-infection. About a third (n = 42, 31%) of the ulcers among the *H. ducreyi* cases were co-infected with other STIs; one-quarter (n = 34, 25%) with *T. pallidum* and 7% (n = 10) with HSV (Table 1).

### Clinical and sexual characteristics

Of the 137 *H. ducreyi* positive participants, 15 had additional clinical data and photographs of lesions obtained after enrollment. Most participants reported having only one sexual partner in the past month (median = 1 [IQR: 1, 1) or 3 months (median = 1 [IQR: 1, 2]) (Table 2). Transactional sex was common: 3/6 (50%) in the past year and 2/9 (22%) in the past month. A third (n = 3/9, 33%) of participants reported alcohol or drug use during last sexual encounter (Table 2).

**Figure 1:**
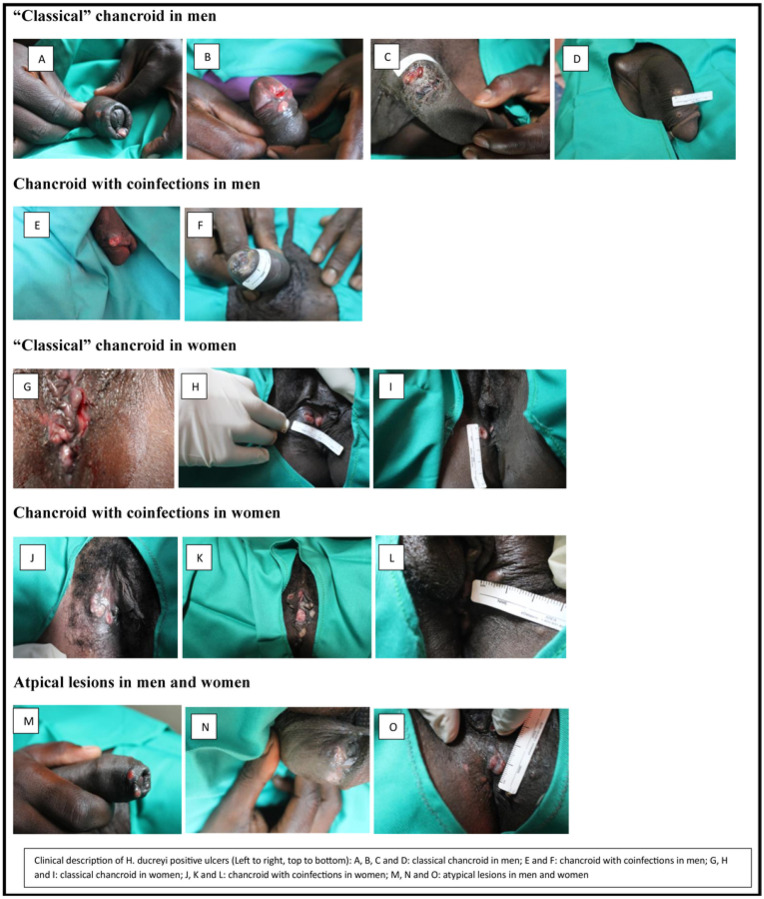
Clinical presentations of chancroid confirmed by *H. ducreyi* PCR

The characteristics of the 15 participants with chancroid and additional clinical data are summarized in Table 3. The median duration of ulcers was 14 days (IQR: 7, 25) prior to presentation. Over half had multiple ulcers (n = 8, 53%), which were commonly located on the penis among male participants (n = 8/8, 100%) and vulva/labia/vagina (n = 5/7, 71%) among female participants. Few (n = 3/15, 20%) had adenopathy, but most ulcers (n = 8/9, 89%) were associated with pain, among those assessed. The presentations of chancroid included ulcers with both regular and irregular borders, with raised ragged borders and some induration (Fig. 1). The ulcers were reported to have either a purulent or erythematous base, and some were easy to bleed.

## DISCUSSION

We reviewed the demographic and clinical characteristics of patients with *H. ducreyi* genital ulcers confirmed by PCR in Malawi and found that a third of our participants were co-infected with other STIs and a fifth had HIV infection. We had limited sexual history data to adequately characterize the epidemiology of chancroid in Malawi, but among participants with additional questionnaires, a third reported a history of transactional sex and a third reported alcohol or drug use during the last sexual encounter. Based on these data, the presence of co-infections contributing to GUD, and transmission among high-risk individuals may be contributing to the persistence of chancroid in this community.

We found a range of clinical presentations of chancroid GUD among participants, consistent with the literature.^1,2^ The clinical picture was mixed among both men and women and convoluted by co-infections. For instance, among classical chancroid ulcers, the shapes, depths, borders and surfaces had mixed presentations (regular and irregular, deep and flat, indurated and non-indurated, ragged and smooth), which explains the suboptimal sensitivity and specificity of clinical diagnosis.^11^

Overall, we found that co-infections were common in nearly a third of persons with chancroid, with syphilis as the most common - although this could have partly been due to the fact that the study by Matoga, et al. deliberately recruited patients suspected of primary syphilis.^3^ Correlation analysis reported from a review of GUD epidemiological patterns in South Africa identified strong links between *H. ducreyi* and HSV-1 (0.64) and between *H. ducreyi* and *C. trachomatis* (0.56), underscoring the importance for integrated diagnostic strategies.^14^ The presence of coinfections could represent shared transmission patterns among high-risk individuals, in which the likelihood of transmission is potentiated for both pathogens.

A systematic review on infections caused by *H. ducreyi* noted that highly sexually active individuals such as sex workers can serve as clinical reservoirs of *H. ducreyi* infections in the community.^1^ Mass antimicrobial prophylaxis of sex workers has led to reduction of chancroid cases in the past two decades in formerly endemic areas.^1^ For instance, in Thailand, presumptive treatment of sex workers with quinolones and 100% condom use led to a 95% reduction in chancroid cases in the 1990s.^15^ In a South African mining community, presumptive treatment with azithromycin (1 g, oral, given monthly for 3 months), condom use and awareness led to a 85% decrease in genital ulcers in sex workers and a 78% decrease in the community over a 9 month follow up period.^16^

In Malawi, it is unknown if similar populations of highly sexually active individuals could be clinical reservoirs of *H. ducreyi* infections. Although we did not have sexual histories on the majority of our participants with chancroid, 22–50% of participants who were asked regarding exchange of sex for goods/money/favors reported transactional sex in the past month or the past year. Nonetheless, given that asymptomatic infection with *H. ducreyi* is rare – identified in 2% of cervical swabs of sex workers without genital ulcers in Gambia^17^ - further research to understand the transmission dynamics in Malawi from symptomatic or asymptomatic highly sexually active individuals such as sex workers is vital.

Considering that the sustained reduction in chancroid cases across the world has been attributed to mass antimicrobial prophylaxis and syndromic management particularly in LMICs,^1^ it is surprising why chancroid cases in Malawi have persisted despite syndromic management which includes chancroid treatment since the early 1990s. The current World Health Organization (WHO) guidelines for syndromic management recommend including ciprofloxacin in the treatment of GUD only in geographical settings where chancroid cases are reported or emerging.^18,19^ In Malawi, ciprofloxacin has been included in syndromic therapy for all GUD cases. Unfortunately, no antibiotic resistance studies for *H. ducreyi* have been conducted in recent years in Malawi;^3^

Although we focused on GUD cases, *H. ducreyi* has been reported as a cause of cutaneous ulcers (CU) since 2006, likely resulting from entry through minor traumatic wounds in asymptomatically colonized skin among the lower limbs of children and adults who migrated from yaws-endemic regions.^1^ *H. ducreyi* CU strains have a distinct ecologic niche, but it is evident that they evolved from GUD strains, hence sharing a core genome and other biological properties.^1^ There have been no studies in Malawi to determine if CU cases are due to chancroid in children or adults. Determining the role of *H. ducreyi* CU strains and their molecular relationship with GUD strains using whole genome sequence (WGS) analyses may also shed some light in understanding the epidemiology and persistence of chancroid in Malawi. In addition, WGS could be utilized to investigate possible correlations between genetic resistance markers with *in vitro* antimicrobial susceptibility testing and clinical data; this could assist in future studies to determine whether antimicrobial resistance to ciprofloxacin is contributing to ongoing transmission in the area.

This review has some limitations. Since one of our studies deliberately recruited 40% of participants with HIV infection^6^ and the other recruited patients suspected of primary syphilis,^3^ this may have resulted in inflation of co-infection rates with HIV and syphilis. The number of *H. ducreyi* confirmed cases by PCR with additional clinical data and photographs was inadequate to determine if there were any unique changes in the clinical presentation of *H. ducreyi* GUD in Malawi. In addition, the number of cases with additional social and sexual history data was also insufficient to generate generalizable data on the epidemiology and reservoirs of infection. Although the two studies in this review were conducted around the same time, the molecular assays used in the diagnosis of *H. ducreyi* were different and only one study had external validation of *H. ducreyi* results at the WHO collaborating Center for Gonorrhea and other STIs.

Despite these limitations, this review demonstrates the high and persistent prevalence of *H. ducreyi* GUD, and the different clinical manifestations of contemporary chancroid. It confirms the complexity in clinical diagnosis of chancroid due to various presentations and high level of co-infections with other GUD etiologies. Additional studies to investigate the genomics and antimicrobial resistance of circulating *H. ducreyi* may help explain the epidemiology and transmission networks in Malawi. Future research involving *H. ducreyi* will be vital in planning public health efforts to reduce the morbidity in Malawi and in the development of interventions for possible eradication of chancroid in regions where it remains prevalent.

## Supplementary Material

Supplementary Files

This is a list of supplementary files associated with this preprint. Click to download.

- TABLEs.docx

## Figures and Tables

**Figure 2:**
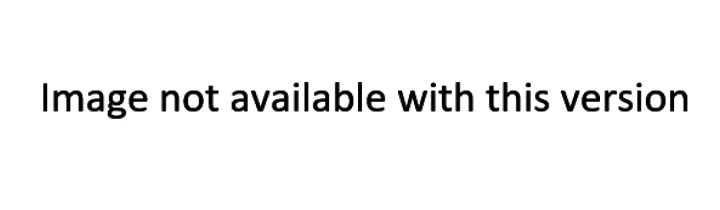
Legend not included with this version
